# Lipidomic data on lipid droplet triglyceride remodelling associated with protection of breast cancer cells from lipotoxic stress

**DOI:** 10.1016/j.dib.2018.03.033

**Published:** 2018-03-12

**Authors:** Eva Jarc, Thomas O. Eichmann, Robert Zimmermann, Toni Petan

**Affiliations:** a Department of Molecular and Biomedical Sciences, Jožef Stefan Institute, Jamova cesta 39, SI-1000 Ljubljana, Slovenia; b Jožef Stefan International Postgraduate School, Ljubljana, Slovenia; c Institute of Molecular Biosciences, University of Graz, Graz, Austria; d Center for Explorative Lipidomics, BioTechMed-Graz, Graz, Austria; e BioTechMed-Graz, Graz, Austria

**Keywords:** Lipid droplets, Lipidomics, Adipose triglyceride lipase, Polyunsaturated fatty acid, Cancer, Phospholipase A_2_

## Abstract

The data presented here is related to the research article entitled “Lipid droplets induced by secreted phospholipase A_2_ and unsaturated fatty acids protect breast cancer cells from nutrient and lipotoxic stress” by E. Jarc et al., Biochim. Biophys. Acta 1863 (2018) 247–265. Elevated uptake of unsaturated fatty acids and lipid droplet accumulation are characteristic of aggressive cancer cells and have been associated with the cellular stress response. The present study provides lipidomic data on the triacylglycerol (TAG) and phosphatidylcholine (PC) composition of MDA-MB-231 breast cancer cells exposed to docosahexaenoic acid (DHA; 22:6, ω-3). Datasets provide information on the changes in lipid composition induced by depletion of adipose triglyceride lipase (ATGL) and by exogenous addition of secreted phospholipase A_2_ (sPLA_2_) in DHA-treated cells. The presented alterations in lipid composition, mediated by targeting lipid droplet biogenesis and lipolysis, are associated with protection from lipotoxicity and allow further investigation into the role of lipid droplets in the resistance of cancer cells to lipotoxic stress.

**Specifications Table**

**Table:** 

Subject area	*Biochemistry and Molecular Biology*
More specific subject area	*Lipidomics*
Type of data	*Tables*
How data was acquired	*Ultra-performance liquid chromatography-quadrupole time-of-flight mass spectrometry (UPLC/qTOF-MS)*
Data format	*Analysed*
Experimental factors	*Analysis of triacylglycerol and phosphatidylcholine species extracted from MDA-MB-231 cells treated with exogenous docosahexaenoic acid, human group X secreted phospholipase A*_*2*_*and depleted in adipose triglyceride lipase*
Experimental features	*Lipidomic analyses of cellular lipids by UPLC/qTOF-MS*
Data source location	*Not applicable*
Data accessibility	*Provided with this article*

**Value of the data**

• This lipidomic data provides information on the lipid composition of Ras-driven aggressive MDA-MB-231 breast cancer cells exposed to nutrient stress [1].
• It identifies changes in membrane and lipid droplet (LD) composition induced by alterations in LD biogenesis and lipolysis and associated with protection from the lipotoxicity of polyunsaturated fatty acids.
• The data describes triacylglycerol (TAG) and phosphatidylcholine (PC) remodelling mediated by human group X secreted phospholipase A_2_ (sPLA_2_) in docosahexaenoic acid (DHA)-treated and adipose triglyceride lipase (ATGL)-depleted breast cancer cells.
• The data describes changes in lipid composition following inhibition of TAG lipolysis and LD breakdown by ATGL depletion in DHA-treated breast cancer cells.
• The data will foster further examination of the role of LDs in the resistance of cancer cells to nutrient and lipotoxic stress.

## 1 Data

In order to determine the changes in TAG composition of LDs and in the profile of cellular PC associated with sPLA_2_-induced protection of breast cancer cells from DHA lipotoxicity, we extracted lipids from MDA-MB-231 cells treated with 100 µM DHA and a combination of 100 µM DHA and 10 nM sPLA_2_ and performed lipidomic analyses [1]. The TAG and PC profiles determined are presented in Table 1, Table 2, respectively.

**Table 1:** TAG profiles of control, DHA- and sPLA_2_-treated MDA-MB-231 breast cancer cells.

**TAG composition (% of total)**^a^			
**TAG species**^b^	Untreated^c^	DHA	DHA+sPLA_2_
**46:0**	0.36 ± 0.03	n.d.	0.05 ± 0.02
**48:0**	1.2 ± 0.1	0.07 ± 0.01	0.16 ± 0.03
**48:1**	1.3 ± 0.1	0.12 ± 0.01	0.2 ± 0.1
**50:0**	3.3 ± 0.4	0.14 ± 0.02	0.4 ± 0.1
**50:1**	5.5 ± 0.1	0.4 ± 0.1	1.0 ± 0.2
**50:2**	2.3 ± 0.2	0.2 ± 0.1	0.6 ± 0.1
**52:0**	6.0 ± 1.0	0.26 ± 0.04	0.8 ± 0.1
**52:1**	11.3 ± 0.3	1.1 ± 0.1	1.8 ± 0.3
**52:2**	8.1 ± 0.2	1.0 ± 0.1	2.0 ± 1.0
**52:3**	3.0 ± 0.3	0.44 ± 0.03	0.9 ± 0.2
**52:4**	0.64 ± 0.03	0.15 ± 0.01	0.3 ± 0.1
**54:0**	1.8 ± 0.1	0.06 ± 0.01	0.17 ± 0.01
**54:1**	8.2 ± 0.3	0.72 ± 0.02	1.3 ± 0.2
**54:2**	6.0 ± 1.0	0.7 ± 0.1	1.3 ± 0.1
**54:3**	5.0 ± 1.0	0.8 ± 0.1	1.7 ± 0.4
**54:4**	2.9 ± 0.1	0.6 ± 0.1	1.1 ± 0.2
**54:5**	2.4 ± 0.1	n.d.	n.d.
**54:6**	1.2 ± 0.1	2.6 ± 0.4	2.6 ± 0.2
**54:7**	0.22 ± 0.03	1.1 ± 0.1	1.26 ± 0.03
**56:0**	1.0 ± 0.1	n.d.	0.10 ± 0.01
**56:1**	1.85 ± 0.02	0.16 ± 0.03	0.23 ± 0.03
**56:2**	1.8 ± 0.1	0.26 ± 0.02	0.4 ± 0.1
**56:3**	1.6 ± 0.1	0.28 ± 0.02	0.43 ± 0.03
**56:6**	4.1 ± 0.3	3.0 ± 1.0	5.4 ± 0.4
**56:7**	1.2 ± 0.2	4.4 ± 0.3	6.7 ± 0.1
**58:1**	1.9 ± 0.1	0.120 ± 0.004	0.25 ± 0.01
**58:2**	1.19 ± 0.02	0.12 ± 0.02	0.23 ± 0.03
**58:3**	0.6 ± 0.1	n.d.	0.17 ± 0.02
**58:5**	1.6 ± 0.2	0.17 ± 0.02	0.7 ± 0.1
**58:6**	2.5 ± 0.2	n.d.	n.d.
**58:7**	1.7 ± 0.2	4.6 ± 0.4	7.2 ± 0.2
**58:8**	0.5 ± 0.1	2.8 ± 0.2	4.8 ± 0.1
**58:9**	0.12 ± 0.02	1.00 ± 0.03	1.7 ± 0.1
**58:10**	n.d.	0.83 ± 0.02	1.3 ± 0.1
**58:11**	0.02 ± 0.01	0.91 ± 0.04	1.1 ± 0.1
**58:12**	0.03 ± 0.02	3.1 ± 0.3	1.9 ± 0.2
**60:1**	2.21 ± 0.04	n.d.	0.31 ± 0.04
**60:2**	1.47 ± 0.03	0.146 ± 0.002	0.31 ± 0.02
**60:3**	0.5 ± 0.1	0.04 ± 0.01	0.16 ± 0.01
**60:4**	0.32 ± 0.04	0.05 ± 0.02	0.12 ± 0.02
**60:10**	0.07 ± 0.01	1.91 ± 0.02	3.0 ± 0.2
**60:12**	0.8 ± 0.1	18.0 ± 1.0	14.1 ± 1.1
**60:13**	n.d.	3.4 ± 0.1	1.6 ± 0.3
**62:1**	0.9 ± 0.1	n.d.	n.d.
**62:2**	0.79 ± 0.03	0.05 ± 0.01	0.163 ± 0.003
**62:3**	0.40 ± 0.02	0.027 ± 0.002	0.112 ± 0.004
**62:11**	n.d.	1.1 ± 0.2	1.5 ± 0.2
**62:12**	0.5 ± 0.1	9.0 ± 0.3	9.0 ± 1.0
**62:13**	0.8 ± 0.1	15.0 ± 1.0	14.0 ± 1.0
**62:14**	n.d.	2.9 ± 0.1	2.2 ± 0.2
**66:18**	0.3 ± 0.1	15.0 ± 2.0	7.0 ± 1.0

a TAG composition data is presented as mean percentages of each species per total detected species and normalised to total cellular protein.

b TAG species are described based on the number of carbon atoms and double bonds.

c MDA-MB-231 cells were treated with 100 µM DHA and 10 nM sPLA_2_ in complete medium for 48 h and cell lysates were collected for UPLC/MS analysis. Values are means ± SEM of three independent experiments; n.d., not determined.

**Table 2:** PC profiles of control, DHA- and sPLA_2_-treated MDA-MB-231 breast cancer cells.

**PC composition (% of total)**^a^			
**PC species**^b^	Untreated^c^	DHA	DHA+sPLA_2_
**30:0**	2.7 ± 0.2	4.8 ± 0.3	3.2 ± 0.2
**30:1**	2.3 ± 0.2	2.3 ± 0.1	2.4 ± 0.2
**32:0**	5.0 ± 0.1	21.0 ± 0.3	13.0 ± 1.0
**32:1**	9.0 ± 1.0	7.0 ± 1.0	6.1 ± 0.3
**34:1**	28.0 ± 1.0	17.0 ± 1.0	23.0 ± 1.0
**34:2**	5.3 ± 0.3	2.9 ± 0.2	3.1 ± 0.1
**34:3**	0.26 ± 0.01	1.1 ± 0.1	0.6 ± 0.1
**36:0**	0.47 ± 0.04	0.31 ± 0.02	0.6 ± 0.1
**36:1**	21.0 ± 1.0	7.9 ± 0.3	15.0 ± 1.0
**36:2**	13.0 ± 0.1	4.9 ± 0.1	7.6 ± 0.2
**36:4**	2.46 ± 0.04	2.65 ± 0.01	2.9 ± 0.1
**36:5**	0.48 ± 0.04	1.4 ± 0.1	0.9 ± 0.1
**36:6**	0.07 ± 0.01	1.0 ± 0.2	0.29 ± 0.01
**38:3**	1.9 ± 0.1	1.1 ± 0.1	1.5 ± 0.1
**38:4**	3.0 ± 0.1	2.6 ± 0.3	3.4 ± 0.4
**38:5**	2.0 ± 0.3	2.7 ± 0.2	3.1 ± 0.1
**38:6**	1.1 ± 0.1	9.0 ± 1.0	6.0 ± 1.0
**40:6**	1.5 ± 0.1	7.0 ± 1.0	6.0 ± 1.0
**40:7**	0.5 ± 0.1	3.1 ± 0.3	2.0 ± 0.3

a PC composition data is presented as mean percentages of each species per total detected species and normalised to total cellular protein.

b PC species are described based on the number of carbon atoms and double bonds.

c MDA-MB-231 cells were treated with 100 µM DHA and 10 nM sPLA_2_ in complete medium for 48 h and cell lysates were collected for UPLC/MS analysis. Values are means ± SEM of three independent experiments; n.d., not determined.

In order to determine the changes in TAG composition of LDs and in the profile of cellular PC in ATGL-depleted breast cancer cells, MDA-MB-231 cells were transfected with ATGL-targeting siRNA or non-targeting scrambled siRNA (SCR). In addition, cells were treated with 10 nM sPLA_2_ and 100 µM DHA. Lipids were extracted and lipidomic analyses performed. The data presented includes the detected TAG (Table 3) and PC species (Table 4) in control (SCR) and ATGL-depleted MDA-MB-231 breast cancer cells (siATGL), either untreated or treated with exogenous DHA, sPLA_2_ or both.

**Table 3:** TAG profiles of MDA-MB-231 breast cancer cells depleted in ATGL and treated with exogenous DHA, sPLA_2_ or both.

	**TAG composition (% of total)**^a^					
**TAG species**^b^	SCR^c^	siATGL	SCR+DHA	siATGL+DHA	SCR+DHA+sPLA_2_	siATGL+DHA+sPLA_2_
**46:0**	0.30 ± 0.03	0.42 ± 0.02	0.031 ± 0.003	0.05 ± 0.01	0.03 ± 0.01	0.03 ± 0.01
**48:0**	1.3 ± 0.1	1.9 ± 0.1	0.11 ± 0.02	0.16 ± 0.01	0.08 ± 0.01	0.14 ± 0.01
**48:1**	1.19 ± 0.02	1.5 ± 0.1	0.16 ± 0.02	0.26 ± 0.04	0.15 ± 0.03	0.17 ± 0.03
**50:0**	3.8 ± 0.3	5.41 ± 0.03	0.3 ± 0.1	0.5 ± 0.1	0.2 ± 0.1	0.5 ± 0.1
**50:1**	5.1 ± 0.2	6.8 ± 0.1	0.5 ± 0.1	1.1 ± 0.2	0.6 ± 0.1	0.7 ± 0.1
**50:2**	1.94 ± 0.04	2.4 ± 0.2	0.3 ± 0.1	0.6 ± 0.1	0.3 ± 0.1	0.3 ± 0.1
**52:0**	6.3 ± 0.4	7.9 ± 0.2	0.5 ± 0.1	0.9 ± 0.1	0.4 ± 0.1	0.75 ± 0.02
**52:1**	11.0 ± 1.0	12.0 ± 1.0	1.1 ± 0.2	2.2 ± 0.3	1.1 ± 0.2	1.6 ± 0.1
**52:2**	8.0 ± 1.0	8.9 ± 0.4	1.4 ± 0.1	2.1 ± 0.3	1.3 ± 0.2	1.4 ± 0.2
**52:3**	2.3 ± 0.2	2.7 ± 0.2	0.6 ± 0.1	0.7 ± 0.1	0.5 ± 0.1	0.4 ± 0.1
**52:4**	0.68 ± 0.03	0.7 ± 0.1	0.20 ± 0.03	0.21 ± 0.02	0.21 ± 0.03	0.19 ± 0.02
**54:0**	1.7 ± 0.2	1.75 ± 0.03	0.09 ± 0.01	0.2 ± 0.1	0.07 ± 0.01	0.1327 ± 0.0002
**54:1**	8.1 ± 0.4	8.0 ± 1.0	0.7 ± 0.1	1.3 ± 0.2	0.7 ± 0.1	1.2 ± 0.2
**54:2**	5.5 ± 0.3	5.4 ± 0.2	0.6 ± 0.1	1.3 ± 0.2	0.9 ± 0.1	1.02 ± 0.01
**54:3**	5.0 ± 1.0	5.0 ± 1.0	1.1 ± 0.1	1.5 ± 0.2	1.2 ± 0.1	0.98 ± 0.01
**54:4**	2.5 ± 0.2	2.3 ± 0.1	0.6 ± 0.1	0.7 ± 0.1	0.8 ± 0.1	0.7 ± 0.1
**54:5**	2.1 ± 0.04	1.7 ± 0.1	n.d.	n.d.	n.d.	n.d.
**54:6**	1.4 ± 0.2	1.11 ± 0.04	2.5 ± 0.4	2.9 ± 0.3	2.4 ± 0.4	2.3 ± 0.2
**54:7**	0.21 ± 0.03	0.16 ± 0.02	1.0 ± 0.1	1.1 ± 0.1	1.1 ± 0.1	1.2 ± 0.1
**56:0**	1.0 ± 0.1	1.10 ± 0.02	0.06 ± 0.01	0.11 ± 0.04	n.d.	0.11 ± 0.02
**56:1**	2.0 ± 0.1	1.6 ± 0.1	0.14 ± 0.01	0.25 ± 0.03	0.12 ± 0.02	0.169 ± 0.004
**56:2**	1.9 ± 0.1	1.7 ± 0.1	0.23 ± 0.02	0.40 ± 0.1	0.23 ± 0.03	0.25 ± 0.02
**56:3**	1.50 ± 0.03	1.28 ± 0.03	0.20 ± 0.03	0.34 ± 0.04	0.32 ± 0.04	0.30 ± 0.01
**56:6**	3.9 ± 0.4	2.4 ± 0.04	4.0 ± 1.0	3.6 ± 0.4	5.0 ± 1.0	4.7 ± 0.3
**56:7**	1.7 ± 0.3	1.1 ± 0.1	5.0 ± 1.0	4.6 ± 0.3	6.9 ± 0.4	6.3 ± 0.4
**58:1**	1.91 ± 0.02	1.68 ± 0.04	0.11 ± 0.02	0.21 ± 0.04	0.13 ± 0.02	0.22 ± 0.04
**58:2**	1.2 ± 0.1	1.02 ± 0.01	0.12 ± 0.01	0.20 ± 0.02	0.12 ± 0.02	0.13 ± 0.01
**58:3**	0.62 ± 0.04	0.51 ± 0.03	0.06 ± 0.01	0.11 ± 0.01	0.11 ± 0.02	0.14 ± 0.04
**58:5**	1.7 ± 0.2	0.8 ± 0.1	0.3 ± 0.1	0.26 ± 0.02	0.7 ± 0.2	n.d.
**58:6**	3.0 ± 0.4	1.9 ± 0.3	n.d.	n.d.	n.d.	n.d.
**58:7**	2.1 ± 0.3	1.7 ± 0.4	4.9 ± 0.4	4.9 ± 0.2	7.0 ± 1.0	6.9 ± 0.3
**58:8**	0.9 ± 0.1	0.6 ± 0.2	3.4 ± 0.4	3.4 ± 0.2	5.2 ± 0.2	4.9 ± 0.1
**58:9**	0.2 ± 0.1	0.2 ± 0.1	1.0 ± 0.1	0.92 ± 0.01	1.8 ± 0.1	1.7 ± 0.1
**58:10**	n.d.	0.07 ± 0.03	0.82 ± 0.01	0.78 ± 0.02	1.267 ± 0.002	1.23 ± 0.01
**58:11**	n.d.	n.d.	0.9 ± 0.1	0.88 ± 0.03	1.1 ± 0.1	1.2 ± 0.1
**58:12**	n.d.	0.03 ± 0.01	2.7 ± 0.4	2.9 ± 0.3	2.0 ± 0.4	2.1 ± 0.1
**60:1**	2.3 ± 0.1	1.85 ± 0.02	0.13 ± 0.04	0.22 ± 0.03	0.15 ± 0.03	0.26 ± 0.03
**60:2**	1.50 ± 0.02	1.21 ± 0.02	0.14 ± 0.01	0.20 ± 0.02	0.16 ± 0.02	0.21 ± 0.04
**60:3**	0.60 ± 0.02	0.47 ± 0.02	0.05 ± 0.01	0.10 ± 0.02	0.09 ± 0.01	0.10 ± 0.02
**60:4**	0.4 ± 0.1	0.3 ± 0.10	n.d.	0.05 ± 0.01	0.09 ± 0.02	0.08 ± 0.01
**60:10**	0.1 ± 0.1	0.11 ± 0.04	1.9 ± 0.1	1.76 ± 0.01	3.1 ± 0.2	3.2 ± 0.1
**60:12**	1.1 ± 0.4	0.45 ± 0.03	18.7 ± 0.3	18.0 ± 1.0	15.0 ± 1.0	16.0 ± 0.4
**60:13**	0.1 ± 0.1	0.062 ± 0.004	3.0 ± 0.4	3.1 ± 0.1	2.0 ± 1.0	2.5 ± 0.1
**62:1**	1.0 ± 0.1	0.76 ± 0.03	n.d.	0.08 ± 0.01	n.d.	0.10 ± 0.01
**62:2**	0.9 ± 0.1	0.620 ± 0.003	0.06 ± 0.02	0.08 ± 0.01	0.08 ± 0.02	0.12 ± 0.01
**62:3**	0.43 ± 0.02	0.31 ± 0.01	0.04 ± 0.01	0.050 ± 0.004	0.07 ± 0.02	0.07 ± 0.01
**62:11**	n.d.	0.04 ± 0.01	1.0 ± 0.1	n.d.	1.0 ± 0.1	1.2 ± 0.1
**62:12**	0.32 ± 0.04	0.3 ± 0.1	8.7 ± 0.2	6.6 ± 0.4	8.2 ± 0.1	7.0 ± 1.0
**62:13**	0.5 ± 0.2	0.43 ± 0.01	17.3 ± 1.3	14.6 ± 1.1	15.59 ± 0.02	15.0 ± 1.0
**62:14**	n.d.	n.d.	2.7 ± 0.4	2.6 ± 0.2	2.2 ± 0.4	2.4 ± 0.2
**66:18**	0.18 ± 0.04	0.18 ± 0.01	12.3 ± 2.2	11.2 ± 1.4	7.0 ± 2.0	7.0 ± 1.0

a The lipidomic data is presented as mean percentages of each species per total detected species and normalised to total cellular protein.

b TAG species are presented according to the number of carbon atoms and double bonds.

c MDA-MB-231 cells were reverse transfected with ATGL-targeting siRNA (siATGL) or non-targeting scrambled siRNA (SCR) and treated with 100 µM DHA and 10 nM sPLA_2_ in complete medium for 48 h. Cell lysates were collected for UPLC/MS analysis. Values are means ± SEM of three independent experiments; n.d., not determined.

**Table 4:** PC profiles of MDA-MB-231 breast cancer cells depleted in ATGL and treated with exogenous DHA, sPLA_2_ or both.

	**PC composition (% of total)**^a^					
**PC species**^b^	SCR^c^	siATGL	SCR+DHA	siATGL+DHA	SCR+DHA+sPLA_2_	siATGL+DHA+sPLA_2_
**30:0**	2.6 ± 0.3	2.7 ± 0.2	4.3 ± 0.1	5.7 ± 0.2	2.8 ± 0.1	4.7 ± 0.4
**30:1**	1.9 ± 0.3	2.2 ± 0.2	1.88 ± 0.04	2.44 ± 0.02	1.8 ± 0.1	2.3 ± 0.1
**32:0**	5.3 ± 0.2	5.3 ± 0.2	21.0 ± 1.0	25.0 ± 1.0	12.0 ± 1.0	12.2 ± 1.1
**32:1**	9.0 ± 1.0	9.5 ± 0.4	6.6 ± 0.2	7.3 ± 0.2	5.8 ± 0.1	6.3 ± 0.2
**34:1**	28.0 ± 1.0	28.8 ± 1.4	19.0 ± 1.0	18.0 ± 1.0	25.0 ± 1.0	21.2 ± 0.2
**34:2**	5.4 ± 0.2	4.9 ± 0.4	3.1 ± 0.1	2.9 ± 0.1	3.2 ± 0.1	2.6 ± 0.2
**34:3**	0.26 ± 0.02	0.28 ± 0.01	1.12 ± 0.01	1.2 ± 0.1	0.58 ± 0.03	0.6 ± 0.1
**36:0**	0.38 ± 0.01	0.5 ± 0.1	0.321 ± 0.001	0.29 ± 0.02	0.51 ± 0.03	0.5 ± 0.1
**36:1**	20.0 ± 1.0	21.8 ± 1.4	9.0 ± 0.1	6.0 ± 1.0	15.0 ± 1.0	13.6 ± 1.1
**36:2**	13.4 ± 0.3	12.8 ± 0.3	5.8 ± 0.1	4.3 ± 0.3	9.0 ± 1.0	6.3 ± 0.3
**36:4**	2.4 ± 0.1	2.4 ± 0.1	2.6 ± 0.1	2.8 ± 0.1	2.9 ± 0.2	2.8 ± 0.1
**36:5**	0.5 ± 0.1	0.26 ± 0.02	1.31 ± 0.03	1.8 ± 0.1	0.9 ± 0.1	1.52 ± 0.02
**36:6**	0.11 ± 0.02	0.04 ± 0.01	0.8 ± 0.1	0.8 ± 0.1	0.4 ± 0.1	0.39 ± 0.02
**38:3**	1.8 ± 0.1	1.6 ± 0.1	1.05 ± 0.01	0.8 ± 0.1	1.5 ± 0.1	1.33 ± 0.03
**38:4**	2.8 ± 0.4	2.4 ± 0.2	2.5 ± 0.1	1.9 ± 0.1	3.1 ± 0.2	3.0 ± 0.1
**38:5**	2.0 ± 1.0	1.3 ± 0.1	2.4 ± 0.4	1.9 ± 0.2	3.2 ± 0.2	3.1 ± 0.3
**38:6**	1.7 ± 0.3	0.8 ± 0.1	8.0 ± 1.0	9.0 ± 1.0	6.0 ± 1.0	7.9 ± 0.3
**40:6**	1.5 ± 0.2	1.2 ± 0.1	6.0 ± 1.0	6.0 ± 1.0	5. 0 ± 1.0	7.8 ± 0.3
**40:7**	1.0 ± 0.1	0.31 ± 0.01	3.0 ± 0.1	2.3 ± 0.2	2.5 ± 0.2	2.0 ± 0.1

a The lipidomic data is presented as mean percentages of each species per total detected species and normalised to total cellular protein.

b PC species are presented according to the number of carbon atoms and double bonds.

c MDA-MB-231 cells were reverse transfected with ATGL-targeting siRNA (siATGL) or non-targeting scrambled siRNA (SCR) and treated with 100 µM DHA and 10 nM sPLA_2_ in complete medium for 48 h. Cell lysates were collected for UPLC/MS analysis. Values on the graphs are means ± SEM of three independent experiments; n.d., not determined.

## 2 Experimental design, materials and methods

### 2.1 Materials

Breast cancer cells (MDA-MB-231) and culture medium (RPMI-1640) were obtained from ATCC (USA), fetal bovine serum (FBS), Dulbecco's phosphate-buffered saline (DPBS), TrypLE Select and Opti-MEM from Life Technologies (USA). Docosahexaenoic acid (DHA) was from Cayman Chemical (USA), Lipofectamine RNAiMAX from Thermo Fisher Scientific (USA). Human ATGL-targeting siRNAs and Allstars Negative Control siRNA were from Qiagen (Germany). The recombinant wild-type human group X sPLA_2_ was prepared as described previously [2], [3], [4]. All other chemicals were of at least analytical grade and purchased from Sigma-Aldrich (USA) or Serva (Germany).

### 2.2 Cell culture and treatment

MDA-MB-231 cells were cultured in RPMI-1640 medium in the presence of 10% FBS. Adherent cells were detached using TrypLE Select. Aliquots of stock solutions of DHA in absolute ethanol were stored under argon at −80 °C. Prior to addition to cell culture, DHA was incubated in complete medium for 1 h at room temperature. Cells were seeded in complete medium in 6-well plates at 3 × 10^5^ cells/well. ATGL silencing was performed by reverse transfection at the time of seeding using a 20 nM mixture of two validated siRNAs targeted at ATGL (Qiagen) or 20 nM Allstars Negative Control siRNA (Qiagen). Transfection complexes were generated using 7.5 µl/well of Lipofectamine RNAiMAX and Opti-MEM medium. Reverse transfection was performed according to manufacturer's instructions. Cells were left to attach for 24 h and treated with 10 nM sPLA_2_ and 100 µM DHA for the following 48 h.

### 2.3 Lipidomic analyses of triacylglycerol and phospholipid species

Culture plates were put on ice, the cells washed twice with ice-cold DPBS, collected by scraping in 300 µl lysis buffer (20 mM Tris–HCl, pH 7.4, 2 mM EDTA, 2 µl Halt Protease Inhibitor Cocktail, Thermo Scientific, USA), centrifuged at 4 °C (1000 *g*, 10 min), the pellet resuspended in 150 µl lysis buffer and sonicated on ice. Aliquots of 10 µl were taken for total protein determination using the Pierce 660 nm Protein Assay (Thermo Scientific, USA). Total lipids were extracted twice using chloroform/methanol/water (2/1/0.6, v/v/v) containing 500 pmol butylated hydroxytoluene, 1% acetic acid, and 100 pmol of internal standards (ISTD, 17:0-17:0 PC, 17:0-17:0 PE, 17:0-17:0-17:0 TAG, 17:0 LPC, Avanti Polar Lipids) per sample. Extraction was performed under constant shaking for 60 min at room temperature (RT). After centrifugation at 1000 × *g* for 15 min at RT the lower organic phase was collected, 2.5 ml chloroform added to the remaining aqueous phase and the second extraction performed as described above. Combined organic phases of the double-extraction were dried under a stream of nitrogen and dissolved in 150 µl 2-propanol/chloroform/methanol (7/2/1, v/v/v) for UPLC-qTOF analysis. Chromatographic separation was modified after Knittelfelder et al. [5] using an AQUITY-UPLC system (Waters Corporation), equipped with an ACQUITY BEH C18 column (2.1 × 50 mm, 1.7 µm; Waters Corporation). A SYNAPT™G1 qTOF HD mass spectrometer (Waters Corporation) equipped with an ESI source was used for detection. Data acquisition was done by the MassLynx 4.1 software (Waters Corporation) and lipid classes were analysed with the “Lipid Data Analyzer 1.6.2” software [6]. Data were normalised for recovery and extraction- and ionization efficacy using ISTDs.

### 2.4 Data handling

The acquired lipidomic data was normalised to total cellular protein. The values are presented as mean percentages of each species per total detected species in each sample with standard errors of the mean (SEM) of three independent experiments.
